# Feasibility of Goal Attainment Scaling as a patient-reported outcome measure for older patients in primary care

**DOI:** 10.1186/s41687-023-00615-6

**Published:** 2023-07-24

**Authors:** Paul Stolee, Sara Mallinson, Alison Kernoghan, Meaghan Brierley, Catherine Tong, Jacobi Elliott, Lama Abdallah

**Affiliations:** 1 School of Public Health Sciences, University of Waterloo, 200 University Avenue West, Waterloo, ON N2L 3G1 Canada; 2 Department of Community Health Sciences, University of Calgary, Calgary, AB Canada; 3 Health Systems Evaluation and Evidence, Alberta Health Services, Calgary, AB Canada; 4 Lawson Health Research Institute, London, ON Canada

**Keywords:** Goal Attainment Scaling, Patient-reported outcomes, PROMs, Primary care, Geriatrics, Care planning

## Abstract

**Background:**

Goal Attainment Scaling (GAS) is an outcome measure that reflects the perspectives and experiences of patients, consistent with patient-centred care approaches and with the aims of patient-reported outcome measures (PROMs). GAS has been used in a variety of clinical settings, including in geriatric care, but research on its feasibility in primary care practice has been limited. The time required to complete GAS is a barrier to its use by busy primary care clinicians. In this study, we explored the feasibility of lay interviewers completing GAS with older primary care patients.

**Methods:**

Older adults were recruited from participants of a larger study in five primary care clinics in Alberta and Ontario, Canada. GAS guides were developed based on semi-structured telephone interviews completed by a non-clinician lay interviewer; goals were reviewed in a follow-up interview after six months.

**Results:**

Goal-setting interviews were conducted with 41 participants. GAS follow-up guides could be developed for 40 patients (mean of two goals/patient); follow-up interviews were completed with 29 patients. Mobility-focused goals were the most common goal areas identified.

**Conclusions:**

Study results suggest that it is feasible for lay interviewers to conduct GAS over the telephone with older primary care patients. This study yielded an inventory of patient goal areas that could be used as a starting point for future goal-setting interviews in primary care. Recommendations are made for use of GAS and for future research in the primary care context.

## Background

Patients’ priorities and needs are central to the provision of quality care [1, 2]. Patient-centred or person-centred care aims to emphasize the role of the patient in their healthcare journey and to place their needs, opinions, and involvement at the centre of care planning and decision-making [3]. This can be supported by increasing the participation of patients in care planning and by supporting the implementation of self-management initiatives to increase the control that a patient has over their care [4]. Collaborative goal setting can increase the participation of patients and caregivers and facilitate their engagement in self-management [4]. It can also promote trust between providers and patients (and their caregivers) because providers are actively seeking the needs and priorities of the patients and incorporating these into their care plans [4]. A shared understanding of goals between providers and patients is associated with better health outcomes and experiences [5–7]. A recent review of goal-oriented care for primary care patients identified elicitation of patient goals as the first step in the process of goal-oriented care, with patients as active partners [8]. While goal-oriented care is recognized as good practice, especially for patients with multiple health conditions, it is often not part of routine care planning [9–11].

Patient-reported outcome measures (PROMs) “directly assess the lived experiences of service users, capturing their perspectives on their health status and essential subjective constructs such as goal attainment, quality of life and social inclusion” [12, p. 57]. Roe and colleagues argue that PROMs should be co-developed and user-selected, with meaningful involvement of patients/people with lived experience in their development and use. Goal Attainment Scaling (GAS) [12, 13] is an approach that can incorporate goals identified by individual patients within a measure co-developed with a clinician or evaluator, in a manner consistent with the aims expressed by Roe and colleagues. GAS is a measurement approach that involves patients selecting personalized goals that are relevant for them and creating individualized scales to measure the attainment of these goals [13, 14]. GAS was originally developed to evaluate community mental health programs [13] but has since been applied in many program evaluation, research, and clinical contexts [15–19], and has been extensively validated [20, 21]. A number of studies have assessed the test-retest or inter-rater reliability of GAS; these studies have primarily focused on the reliability of the GAS follow-up score assessed by clinicians [22, 23]; Stolee et al. [24] and May-Benson et al. [25] also examined the inter-rater reliability of scale construction.

GAS may be a particularly appropriate PROM approach for use with older patients, who often present with multiple, complex and highly individualized health problems which are often not well accommodated with standardized measures [26]. Studies in geriatric care contexts have found GAS to have construct validity [24, 27], to be responsive to change [27–29], and to be a practical approach to guide patient-centred care [19, 30, 31].

Research on the feasibility of GAS specifically for use in primary care for older adults has been limited. Geriatric care settings commonly involve comprehensive approaches to assessment, specialist physicians, and interdisciplinary teams [26], offering greater time and opportunities for careful and comprehensive consideration of patient goals. While primary care settings also serve many older patients, team-based care is limited and, appointment times are shorter [32]. These factors limit opportunities for engaging patients in goal setting and decision-making, which has been identified as a key element of an effective model of care for older complex patients in primary care [33]. The time required to identify and scale individual goals for each patient is recognized as a barrier to implementation of GAS [19, 28, 31–37]. Previous investigations of the feasibility of GAS in primary care have included extensive clinician involvement and complex goal-setting processes. Toto and colleagues [31] conducted a feasibility study of GAS with older adults in geriatric primary care; their goal-setting process involved a lengthy (up to one hour) home visit by a rehabilitation professional. Javadi and colleagues [35] completed GAS with patients at a family health team; goal-setting involved a computerized goal-setting survey completed by volunteers, with follow-up review and discussion by interprofessional teams. Ford and colleagues [11] investigated the feasibility of a goal-setting intervention for patients in primary care (mean age 80.4); their investigation incorporated a modified version of GAS as an aspect of the intervention, which also included extensive practitioner training and a structured goal-setting consultation.

In a number of studies, researchers have investigated the feasibility of lay interviewers (as opposed to medical/clinical interviewers) administering a range of clinical assessments and diagnostic tools (e.g., [38–41]). In many instances (e.g., [38, 39]), lay interviewers were found to be an acceptable mode of data collection, with some training. We were interested to determine whether the GAS process in primary care might be facilitated by non-clinicians, and thus investigated the feasibility of implementing GAS for older patients in primary care using telephone interviews completed by lay interviewers.

## Methods

### Study design

This study was a sub-project of a larger study aimed at enhancing screening and care planning for older adults in team-based primary care practices in Canada; methods for the larger study are reported in detail elsewhere [42]. This study used a sequential mixed method design [43] in which qualitative interviews were used to guide development of GAS follow-up guides which could generate quantitative measures of goal attainment. Data for this study were collected prior to implementation of the study interventions.

### Setting and participants

All participants in this study were part of the larger multi-site study and were recruited from three primary care clinics in Ontario and two Primary Care Networks in Alberta. Patients were included if they were aged 70 or older and living in the community. Individuals living in long-term care homes and patients who had not been rostered with the practice for more than six months were excluded.

Recruitment followed a stratified purposeful sampling approach [44]. Data from the larger study were used to guide selection of participants with varying levels of health or health risk; these included scores on the EQ-5D-5L (the EuroQol Five Dimensions, Five Levels) health status measure [45], a five-point self-rated global health question, and the number of self-reported chronic illnesses. Components of these scores were used to describe each participant’s overall health status as either low risk, medium risk or high risk. Those with low risk health status were defined as those who rated their health as very good or excellent (4 or 5/5), had two or fewer chronic diseases, and an EQ-5D-5L score between 80 and 100. Moderate risk health status participants had rated their health as good (3/5), had two to three chronic diseases, and an EQ-5D-5L score between 60 and 79. Finally, high risk participants were those who rated their health as poor or fair (1 or 2/5), had three or more chronic diseases, and an EQ-5D-5L score between 30 and 59. At each site, we selected two to three patients from each risk group to participate in the study. Demographic characteristics of the participants were collected on a standard data collection form and are provided in Table 1 (all participants) and Table 2 (participants for whom follow-up was completed).

**Table 1:** Demographics of All Participants at Initial Interview

Characteristics	All Participants (n = 41)	High Risk Participants (n = 14)	Moderate Risk Participants (n = 14)	Low Risk Participants (n = 13)
Gender (Male:Female)	24:17	7:7	7:7	10:3
Age (mean; s.d.)	79; 5	79; 6	79; 5	81; 5
Chronic Conditions (mean)	2.9	4.1	2.9	1.8
Receiving Home Care, n (%)	3 (7.3)	2 (14.3)	1 (7.1)	0
Living Alone, n (%)	13 (31.7)	4 (28.6)	4 (28.6)	5 (38.5)
Caregiver Support, n (%)	9 (22.0)	5 (35.7)	2 (14.3)	2 (15.4)
High school education or less, n (%)	13 (31.7)	7 (50.0)	3 (21.4)	3 (23.1)
Post-Secondary Education, n (%)	28 (68.3)	7 (50.0)	11 (78.6)	10 (71.4)
North American or European Origin, n (%)	40 (97.6)	14 (100)	13 (92.9)	13 (100)

**Table 2:** Demographics of Follow-up Participants

Characteristics	All Participants (n = 29)	High Risk Participants (n = 9)	Moderate Risk Participants (n = 8)	Low Risk Participants (n = 12)
Gender (Male:Female)	19:10	5:4	5:3	9:3
Age (mean; s.d.)	80; 5	82; 5	81; 5	82; 5
Chronic Conditions (mean)	2.7	4.2	2.4	1.8
Receiving Home Care, n (%)	3 (10.3)	2 (22.2)	1 (12.5)	0
Living Alone, n (%)	10 (34.4)	3 (33.3)	2 (25.0)	5 (41.7)
Caregiver Support, n (%)	6 (20.7)	3 (33.3)	1 (12.5)	2 (16.7)
High school education or less, n (%)	10 (34.4)	4 (44.4)	3 (37.5)	3 (25.0)
Post-Secondary Education, n (%)	19 (65.5)	5 (55.6)	6 (75.0)	8 (66.7)
North American or European Origin, n (%)	28 (96.5)	9 (100)	7 (87.5)	12 (100)

### Goal Attainment Scaling procedure

**Fig. 1:**
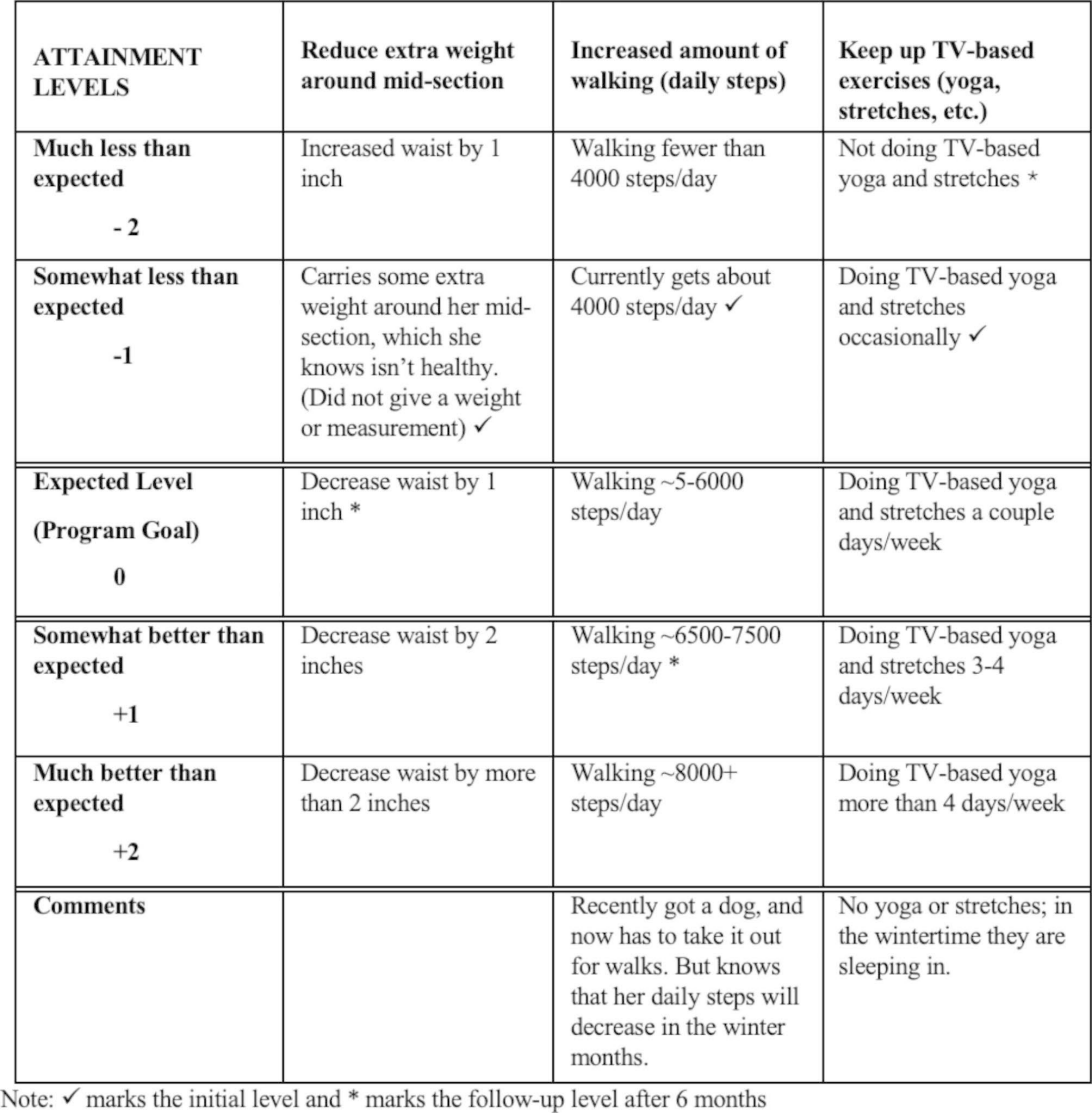
Sample GAS Follow-up Guide

One GAS follow-up guide was created for each patient (“follow-up guide” is the term commonly used to describe the form completed upon initial assessment and then used to guide follow-up assessments). A GAS follow-up guide consists of a table in which the goals identified by each patient are scaled in terms of their level of attainment. Goal attainment is scored on individualized five-point scales, from − 2, much less than expected, to + 2, much better than expected. The expected level of goal attainment, or program goal, is scored as 0. GAS differs from other individualized goal setting tools (e.g., the Canadian Occupational Performance Measure [46]) in that the expected outcomes are defined and operationalized on an *a priori* basis. A sample follow-up guide is provided in Fig. 1.

GAS follow-up guides were created based on individual interviews completed with each patient; interviews were conducted by members of a non-clinician research team. Before interviews began, all interviewers received comprehensive training on constructing GAS guides; training included team members constructing follow-up guides based on case scenarios. Training was led by a researcher (PS), with extensive experience using GAS in clinical settings and research studies, and included a two-hour training session and an in-depth written guide that detailed how to identify goal topics and set attainment levels with participants. The training was delivered to researchers from all sites and the methods and techniques outlined in the guide were consistently applied during all interviews. Consistency in goal-setting was also aided by weekly meetings between the Alberta and Ontario teams. The first set of patient interviews was completed within a month of the initial clinic appointment during which patients were recruited. The interview followed a semi-structured interview guide with the first portion (approximately two-thirds of the interview) related to general study questions about care experiences (completed as part of data collection for the larger study), and the remaining portion related to health goals and priorities of the patient. The language used while questioning and prompting participants was neutral in order to limit any bias in patient responses. The four interviewers (two in Alberta, two in Ontario) are lay, non-clinicians, and Master’s or PhD-level trained qualitative researchers. After the goals were recorded, interviewers verbally confirmed the wording of these with participants to ensure they were an accurate representation of the patient’s goals. All interviews were recorded and transcribed verbatim.

Two GAS guides were constructed for a sample of patients (n = 8) from three sites (in Ontario), as a check on the inter-rater reliability of GAS guide construction, including goal-identification and scaling, similar to the approach used by Stolee et al. [24] and May-Benson et al. [25]. One guide was developed by the interviewer and a second guide was developed by a second researcher based on the audio-recording and transcript. The researchers then reviewed both GAS guides to identify similarities and differences and agreed on one consensus guide to be used for follow-up. In the two primary care networks in Alberta, the interviewer took the lead on drafting the GAS guides and this was then independently checked by two members of the study team. Researchers connected with patients for a follow-up interview after six months to discuss their progress on their goals.

### Assessment of feasibility

As this was an initial exploratory study, our criteria for feasibility were limited:

1. Individual patient goals can be elicited from older primary care patients, at varying levels of health or risk, in a telephone interview conducted by a lay interviewer;
2. Two laypersons will record similar goals based on the same patient interview;
3. The interview will generate an average of three goals per patient; and.
4. Patients can be followed up to review their goals, after six months.

While not explicitly a feasibility criterion, we were interested in the types of goal areas identified, to allow comparison with goal areas identified in other applications of GAS with older patients (e.g., [11, 19, 33, 34]) and to provide an inventory of goals that might serve as a starting point for future applications of GAS in primary care.

To provide data that might be helpful for future research or clinical applications of GAS in primary care, we intended to calculate the means and standard deviations of the initial and follow-up GAS scores. We did not anticipate statistically significant differences between initial and follow-up scores, as no interventions other than usual practice were implemented during this period.

### Statistical analysis

GAS scores were calculated for each patient using their initial and follow-up goal attainment levels. The formula used to calculate GAS scores produces an overall score adjusted for the number of goals, the expected inter-correlation of the goal scales (conventionally assigned a value of 0.3), and the relative weighting of the goals [13]. Current practice favours assigning goals equal weights [47]; this simplifies the calculation of the GAS score, which can then be taken from a published scoring key [48]. The GAS formula generates a T-score; scores for a sufficiently large sample are expected to form a normal distribution with a mean of 50 ± 10. Microsoft Excel was used for organizing and analyzing data.

### Qualitative analysis of administration observations & notes

After the initial and follow-up interviews, interviewers recorded their observations and experiences in field notes; some field notes included verbatim quotes from participants, which were captured during administration of the GAS. Field notes and team debriefs also captured the interviewers’ experiences and impressions during administration of the instrument. These data were entered into NVivo 12, and analyzed inductively; the identification of emergent themes was supported by team discussions.

### Ethics

Prior to the interview, all participants provided informed consent. This study received ethics clearance from the offices of research ethics at the University of Waterloo and the University of Calgary.

## Results

In total, 41 patients across the five primary care networks were recruited and interviewed. Forty GAS guides were created with a total of 85 goals (one to four goals/patient; mean = 2.1 goals/patient); one patient from the moderate risk category was unable to identify a goal and thus was not followed through the rest of the study. Of the 40 patients who identified goals, 29 were successfully contacted after 6-months to review their goals and determine a follow-up GAS score. Participants who did not complete a follow-up interview either could not be reached by telephone, after at least two attempts (n = 8), or had a change of circumstances (new caregiving duties that took up spare time; communication issues due to worsening chronic disease) that caused them to withdraw from the study (n = 2); one participant had died. The mean duration of the complete initial interview (including the portion completed for the larger study) was 19 min (min = 9; max = 47). In general, the last third of the interview (avg: 5:38 min, min: 1:40 min, max 15:00 min) was used to discuss health goals and develop the GAS guide. Brief follow-up interviews consisted only of GAS discussions and lasted a mean of three minutes (min = 1; max = 4).

There was complete agreement between the interviewer and second researcher in Ontario with the identified goal areas; discussion resulted in minor changes in wording and scale definitions on two of the eight guides. In Alberta, the team verification of the GAS guide resulted in very minor wording changes, suggesting good consensus in goal expression and scaling. Of the 29 participants who completed a follow-up GAS guide, 12 were low-risk, eight were moderate-risk, and nine were high-risk. Between one and four goals were set for these followed up patients (mean of two goals/patient), thus the number of goals set was similar to that for the full sample. Mobility-focused goals were the most common, with 27 (31.8% of total goals) goals addressing exercise, step count, or rehabilitation. Other goals related to leisure activities (e.g., able to travel in the car for longer periods of time, able to play the piano), nutrition, weight management, instrumental activities of daily living (IADLs) or tasks for living independently (e.g., cooking, house cleaning) [49], pain management, overall health, physical health, medication use (specifically a reduction in medication use), and mental health. Goal types for all participants and for each risk group are shown in Table 3 (all participants at initial interview) and Table 4 (follow-up participants).

**Table 3:** Goal Types for All Participants at Initial Interview

Goal Type	All goals (N = 85)	High risk participant goals (N = 32)	Moderate risk participant goals (N = 26)	Low risk participant goals (N = 27)
Mobility (%)	27 (31.8)	8 (25.0)	8 (30.8)	11 (40.7)
Leisure (%)	11 (12.9)	4 (12.5)	5 (19.2)	2 (7.4)
Weight Management (%)	11 (12.9)	5 (15.6)	2 (7.7)	4 (14.8)
Nutrition (%)	9 (10.6)	5 (15.6)	1 (3.8)	3 (11.1)
Pain Management (%)	8 (9.4)	2 (6.3)	3 (11.5)	3 (11.1)
IADLs (%)	5 (5.9)	1 (3.1)	2 (7.7)	2 (7.4)
Overall health (%)	5 (5.9)	3 (9.4)	2 (7.7)	0
Physical Health (%)	4 (4.7)	3 (9.4)	1 (3.8)	0
Medications (%)	3 (3.5)	0 (0)	1 (3.8)	2 (7.4)
Mental Health (%)	2 (2.4)	1 (3.1)	1 (3.8)	0

**Table 4:** Goal Types for Follow-up Participants

Goal Type	All goals (N = 58)	High risk participant goals (N = 20)	Moderate risk participant goals (N = 14)	Low risk participant goals (N = 24)
Mobility (%)	21 (36)	6 (30)	4 (29)	11 (46)
Leisure (%)	8 (14)	4 (20)	2 (14)	2 (8)
Nutrition (%)	6 (10)	3 (15)	1 (7)	2 (8)
Weight Management (%)	6 (10)	1 (5)	1 (7)	4 (16.7)
IADLs (%)	5 (9)	1 (5)	2 (14)	2 (8)
Pain Management (%)	5 (9)	1 (5)	2 (14)	2 (8)
Overall health (%)	4 (7)	3 (15)	1 (7)	0
Medications (%)	3 (5)	0	1 (7)	2 (8)

The mean initial GAS score for all patients was 43.6 ± 7.2. For patients who completed follow-up interviews, the mean initial GAS score was 43.9 ± 7.5; the mean follow-up score was 45.9 ± 11.7. Pre-post change in goal attainment score (mean change of 2.4 ± 13.4) was not statistically significant (p = .37) in this pre-intervention phase of the study (i.e., none of the planned study interventions had yet been implemented, so any change in goal attainment would have resulted from “usual care”, patient-initiated actions, or the passage of time). Change in GAS score varied widely over the follow-up period, ranging from − 20.0 to + 41.1 points, suggesting that some patients experienced substantial gains or declines in their identified goals. The mean initial, follow-up, and change in GAS scores for patients in the low, moderate and high risk categories are depicted in Table 5. There was modest upward (but not statistically significant) movement between initial and final GAS scores for the low and high risk patients; the initial and follow-up scores for moderate risk patients were similar (mean change of -0.6 ± 12.6). Compared to the high and low risk participants, moderate risk participants had fewer goals overall (14), and slightly fewer goals per patient (1.8/patient, compared to 2.2 and 2.1, respectively). The types of goals identified by the moderate risk participants differed from the other groups, with a lower proportion of mobility goals and a higher proportion of goals in the IADL, pain management, and medication categories.

**Table 5:** Summarized GAS results and significance of findings

Frailty Risk	Mean number of goals/patient (min, max)		Initial GAS Score (SD)		Follow-up GAS Score (SD)	Change (SD)	p-value
	All patients	Follow-up patients	All patients	Follow-up patients			
**All**	2.2 (1, 4)	2.0 (1, 4)	43.6 (7.2)	43.9 (7.5)	45.9 (11.7)	2.4 (13.4)	0.37
**Low**	2.1 (1, 3)	2.1 (1, 3)	44.0 (7.5)	44.1 (7.8)	46.6 (12.8)	2.5 (13.9)	0.54
**Moderate**	2.0 (0, 4)	1.8 (1, 4)	45.0 (7.1)	45.9 (6.2)	45.2 (13.6)	-0.6 (12.6)	0.89
**High**	2.3 (1, 4)	2.2 (1, 4)	41.9 (7.4)	42.1 (8.5)	46.4 (10.1)	4.4 (13.9)	0.37

### Observations and reflections on the administration of GAS

In Table 6, below, we summarize our team’s reflections and lessons learned from our approach to completing GAS guides with older adult participants, over the phone, using non-clinician interviewers. We found that robust and consistent training across sites was essential; there were limitations to completing this work over the phone; patients often struggled with the notion of “health goals”; and, that it was important to be mindful of potential sensitive goals.

**Table 6:** Reflections on the administration of GAS

***Training & Consistency***: • Our team of interviewers found that the training provided to the data collection team at the start of the study was effective, and that the training guide provided was useful. • Interviewers also commented on the importance of thorough training for multiple teams constructing GAS guides, as well as ongoing communication among them, to ensure that all team members are approaching their GAS guides and data collection consistently. • Core team members facilitated regular communication between sites. • The GAS guide verification processes adopted in Alberta and Ontario suggested good alignment between different goal-setters.	
*Mode of Data Collection*: • Video calls and/or in-person administration may have enhanced this process, particularly when discussing sensitive topics. Feelings and sensitivities of patients are often revealed through their body language and facial expressions, and this was harder to read and/or respond to over the telephone. • Interviewers enjoyed the opportunity to re-connect with participants at two timepoints, and participants often recognized the team members’ voices and remembered the initial GAS guide conversation. In some instances, this led to feelings of rapport	
*Duration and Prefacing the Goals Discussion*: • Baseline interviews were an average of 19 min with the final third of this time specifically devoted to goal setting. • It took a relatively small amount of time to have meaningful discussions with patients about their goals; however, some interviewers found it challenging to work with participants to identify goals. A common initial response was “I don’t have any goals.” • It was helpful to preface the discussion of goals with a question about health experiences and problems, and giving examples of the types of goals they might have. • Because the concept of “health care goals” was confusing to many participants, we often rephrased the question to “if there was something about your health or health habits that you could work on in the coming 6 months, what would it be?”. • By asking about health and experiences first, participants were able to base some of their goals on the health care experiences and issues described in their answers to earlier questions. • Thus, while relatively little time was usually required for the goal-setting discussion, this process benefited from the earlier discussion of health experiences.	
***Discussing Sensitive Topics.*** • Patients seemed more comfortable discussing physical activity, blood pressure, and other measurements, but some seemed hesitant to discuss specifics related to mental health, weight goals and other more personal goals. • In an attempt to decrease the invasiveness of such discussions, we pre-emptively stated reassurances that we did not require specifics that they weren’t willing to share (e.g. their weight, the names of medications that they are on, etc.). They could, alternatively, state something like “My goal is to lose 10 lbs” (rather than sharing their weight), or “I would like to reduce the number of prescriptions that I am taking” (without revealing which medications they were on).	

## Discussion

Processes for collaborative goal-setting with patients are the focus of ongoing research. Various approaches have been explored. Several studies have described primary care clinicians administering goal-setting sessions with patients (e.g., [11, 50, 51]). While these approaches have merit, they require the participation and time of busy clinicians which may not always be available. Boeykens et al. [8] concluded their review of goal-oriented care with a recommendation for further research on “how and what goals are set by the patient” and “how this knowledge could be translated into a tangible workflow”. Our study yielded insights into these two questions, with a focus on the use of GAS for older patients in primary care. If GAS can be feasibly administered by non-clinicians to facilitate goal identification by patients in primary care, at least as an initial step in a goal setting process, this may alleviate some concerns about the time that may be required to incorporate GAS as a PROM in routine clinical practice.

This study suggests that GAS can be used by non-clinicians to facilitate the selection of goals by older primary care patients and for measuring their progress over time. All but one (moderate risk) patient was able to identify at least one personal goal related to their health; 76% were able to identify more than one goal. Thus, it is possible for community-dwelling older adults to develop personal goals using the GAS procedure. While mean change in goal attainment was not statistically significant, nor expected given that there was no intervention, our results indicate that many patients experienced change in their goal attainment levels. Other studies have suggested an association between the act of personal goal setting and the motivation to achieve goals [31, 52, 53].

We found good agreement between laypersons on the goals that were set based on the same patient interview. As displayed in Tables 4 and 5, the most common goal areas were mobility, leisure, nutrition, and weight management. In total, 31.8% of the developed goals focused on mobility. The second most common goal category was leisure, which represented 12.9% of the goals. This is consistent with some previous studies that used GAS with older adult patients, which also identified mobility and leisure as common goals in this population [54]. Toto et al. [31] reported that 50% of goals in their study related to leisure; mobility and other self-care goals were less common. In the study conducted by Ford et al. [11], in which clinicians led goal-setting consultations, management of chronic conditions was a common goal, this may have resulted from a greater clinical emphasis in these interviews. Mobility goals (including walking and balance) were also common. Leisure goals were not specifically identified, but may have been captured in “maintain interests”, “social participation” and “gardening-related” categories. We note that Haladay et al. [55] used qualitative methods (focus group interviews) to develop an inventory of important and measurable goals with the intention of using this inventory to provide prompts to assist patients in identifying GAS goals in the context of outpatient physical therapy for low back pain. In our study, such a list was identified through individual interviews, but we suggest that our inventory of goals (Tables 4 and 5), could be used similarly as a starting point for future goal-setting interviews in primary care, potentially making the process more time-efficient. Haladay and colleagues [55] saw that GAS had advantages over standardized fixed-item PROMs in its ability to reflect goals of importance to patients. This is consistent with the recent findings of Lauritzson and colleagues [56] that the individualized Canadian Occupational Performance Measure was more responsive to clinically important change than the fixed-item PROM used as a comparator.

Although many participants were able to improve their GAS score between initial and follow-up interviews, the individual change varied widely, with some scores showing decline, some remaining the same, and some showing improvement. This variation could be a result of the different amount and types of goals identified among the three groups. IADL goals had an average negative GAS change of about − 7.83 points, while mobility goals had an average positive GAS change of 5.03; this could contribute to the discrepancy in mean score change between these patient groups.

Kiresuk, Smith and Cardillo [14] recommended the identification of at least three GAS goals/patient for optimal reliability, which was the reason we selected three goals as our target. While fewer goals/patient (mean of 2.1) were identified in this study, a target of three may not have been necessary or realistic. McGarrigle et al. [57] suggest that the use of one-goal and two-goal GAS can be clinically responsive, although that research group found that one-goal GAS is ultimately less responsive to clinical interventions than multi-goal GAS [17]. The primary care goal-setting process studied by Ford et al. [11] set between one to three goals/participant with a mean of 2.3 goals, similar to our results.

Training has been long recognized as important in successful use of GAS, although how this is done is often not reported [58, 59]. Our team of researchers received training from a recognized GAS expert (PS), and we had strong alignment between different goal-setters. This success in GAS scale construction may be primarily attributed to good training, consistent processes for interviewing and documentation, ongoing team discussions related to administration of GAS guides, and confirmation of the wording of goals by the patients themselves. We suggest that some review process to confirm GAS guide construction is worthwhile, but likely needed only in the initial learning or implementation stages (and perhaps intermittently thereafter).

While our team completed the interviews over the phone, using video calls would make it easier for interviewers to read body language and provide comfort, support, and empathy when difficult topics are being discussed. In our ongoing work and research with older adults, we have found that many are comfortable using video-conference software [60]. The COVID-19 pandemic prompted the increased use of technology of older adults, so more older adults are likely to be confident in the use of video-conferencing software [61]. In-person interviews would also have advantages, though would require additional time and resources, as was found by Toto et al. [31].

Finally, drawing on our teams’ experience, we emphasize the need for GAS interviewers to have a strong sense of empathy and sensitivity – a need that has been suggested in previous studies [62]. We recommend that data collectors approach these conversations with tact and sensitivity, and that they highlight the potentially sensitive nature of GAS discussions in recruitment materials and interview introductions.

Interviews can have an impact on relationship building with patients, and consequently the rapport needed to set meaningful goals. Mallinson [63] has described the “interactional strangeness” of standardised survey interviews which can limit sense making, potentially constraining effective communication. Nevertheless, as Greenhalgh and colleagues [64] note, standardized interviews, particularly with the objective of creating individualized PROMs, can still support better communication with patients. Lewis and colleagues [65] have proposed that Goal Attainment Scaling be used in combination with motivational interviewing in a process to set and monitor client-centred goals.

### Strengths & limitations

Our study has a number of strengths and limitations. Our sample size was small, and most of the participants were of North American or European origin, thus results are not necessarily generalizable to a larger more diverse population. In future GAS studies for older adults, it is recommended that a larger and more diverse sample of older adults is recruited. We were unable to contact 11/40 patients for follow-up interview. More sustained efforts may have resulted in lower attrition, but we note that our attrition rate was fairly close to that experienced by Ford et al. [11], who were unable to obtain follow-up data on 11/52 participants.

The use of GAS by a non-clinician interviewer rather than by a clinician may make regular use of GAS more feasible in that it could be done routinely by non-clinical members of a primary care team. A strength of this study was that, due to our use of telephone interviews, we were able to recruit patients from a larger geographic region. This was more cost-effective and avoided the efforts that would have been required for participants to transport themselves to a clinic. Although the personal nature of GAS discussions may have been more comfortable in-person, it was feasible to conduct interviews by phone. By checking-in and re-interviewing the participants over time, relationships between the participants and the interviewers were developed that could have the potential to create continuity and make the experience more enjoyable for the participant. On the other hand, while we do not have specific reasons for our inability to contact some participants, in some cases calls may not have been returned because these patients did not value the process.

We found that the interviewer and a second researcher constructed similar GAS follow-up guides from the same interview. Comparison of two independent goal-setting interviews might have produced guides with greater differences.

This study involved non-clinician interviewers conducting interviews to facilitate goal-identification by patients, and we were unable to compare these goals with ones that had been identified by clinicians. GAS goals that are developed by regular health care providers may be informed by a stronger clinical understanding of the patient’s history and health conditions, but there is also evidence that clinicians do not always engage patients effectively in decision-making around their care [6]. A goal-setting process involving non-clinicians might add information to the care planning process that might otherwise have been missed, for example in identifying quality of life goals (such as maintaining leisure interests) that might not be considered in a typical clinical assessment. Future research could compare goals set independently by clinicians and non-clinician interviewers; this research could investigate whether patients were more or less comfortable speaking with a non-clinician about their goals than with a clinician.

We characterize GAS as a PROM, as have others [12, 66, 67]. We should note however that others may categorize GAS differently. Reuben and Jennings [37] differentiate GAS from PROMs in terms of the types of goals considered by each approach; they suggest the use of GAS for objective goals and PROMs for subjective goals involving patients’ expressions of their feelings and perceptions. In our view, both approaches can accommodate patients’ reports of objective goals or outcomes as well as their subjective expressions of outcomes such as anxiety or depression. We also acknowledge that GAS could be applied in a manner in which patient perspectives or outcome goals were NOT considered, for example if goals were set by clinicians without consultation with their patients. In our view however, such an application would not realize much of the potential benefits of individualized outcome measurement.

## Conclusions

In terms of the criteria we had set for the feasibility of GAS as a PROM for older patients in primary care, we found that individual goals could be elicited in a telephone interview by a lay interviewer (criterion 1), and that there was good agreement between laypersons on the goals that were set based on the same patient interview (criterion 2). Criteria 3 and 4 – a targeted average of three goals per patient, and follow-up reviews completed after six months - were not completely achieved, though our results (average of 2.2 goals per patient, and 72.5% follow-up) were similar to results achieved by Ford et al. [11]. Our study also yielded an inventory of patient goal areas that could be used as a starting point for future goal-setting interviews in primary care.

Goal Attainment Scaling is a tool that can be used to support engagement in health care decision making in a variety of settings by encouraging patients to take an active role in developing their care plans by identifying their own goals and priorities. Overall, we found promising evidence of the feasibility of administration of GAS in primary care by non-clinician interviewers, as well as limitations and a need for further research in this context. Building on the results of this study, we see potential applications of GAS as a PROM in both clinical practice and clinical research. In clinical practice, completion of GAS by non-clinician interviewers may facilitate greater engagement of older patients in goal setting and decision-making in primary care settings where the time available for health care professionals to participate in these activities is constrained. A telephone-based approach holds promise with the experience of the COVID-19 pandemic in mind. Clinical research applications could include use of GAS to gain insight into the goals and priorities of older patients and to assess the effectiveness of treatment approaches aimed at achieving these outcomes. In this study, we found GAS to be feasible with older patients at varying levels of health risk, but we note that patients seemed more comfortable discussing some types of goals (e.g., physical activity) than others (e.g., mental health). Future research should continue to investigate for which older patients and treatment goals GAS might be best targeted in primary care settings.

## Data Availability

Under the terms of the ethics clearance received for this project, our data are unavailable for sharing.

## List of Abbreviations

EQ-5D-5L: The EuroQol five dimensions, five levels
GAS: Goal Attainment Scaling
PROMs: Patient reported outcome measures
